# Clinical and molecular characterization of 14 Egyptian children with fructose-1,6-bisphosphatase deficiency

**DOI:** 10.1186/s13052-025-02146-w

**Published:** 2025-12-01

**Authors:** Rofaida M. Magdy, Abdelrahim A. Sadek, Shimaa B. Hemdan, Ahmed S. Mahmoud, Nada H. Abdel Fattah, Elsayed Abdelkreem, Rania G. Abdelatif

**Affiliations:** 1 Metabolic and Genetic Unit, Department of Pediatrics, Faculty of Medicine, Sohag University, Sohag, Egypt; 2 Department of Medical Biochemistry, Faculty of Medicine, Sohag University, Sohag, Egypt; 3 Department of Clinical Pathology, Faculty of Medicine, Sohag University, Sohag, Egypt; 4 Department of Medical Genetics, Faculty of Medicine, Ain Shams University, Cairo, Egypt; 5 Department of Pediatrics, Faculty of Medicine, Sohag University, Nasser, Sohag, 82524 Egypt; 6 Department of Clinical Sciences, Al Rayan National College of Medicine, Al Madinah Al Munawarah, Saudi Arabia

**Keywords:** FBP1, Lactic acidosis, Ketotic hypoglycemia, Variant, Egypt

## Abstract

**Background:**

Fructose-1,6-bisphosphatase (FBP1) deficiency is a rare inherited disease characterized by recurrent episodes of lactic acidosis and ketotic hypoglycemia. To date, no cases have been reported in the Egyptian population. This study aimed to elucidate the phenotypic and molecular spectrum of FBP1 deficiency in Egypt.

**Methods:**

This observational study included children with FBP1 deficiency diagnosed and managed at an Egyptian medical center between 2022 and 2024. Clinical and laboratory data of acute metabolic episodes were thoroughly reviewed. All patients underwent blood acylcarnitine assay, urinary organic acids analysis, and whole-exome sequencing. Patients’ outcomes were classified into favorable, neurodevelopmental impairment, and death.

**Results:**

This cohort included 14 Egyptian children (from 11 families) with FBP1 deficiency. The median age at disease onset was 13 months, ranging from the first week of life to 36 months. All patients exhibited acute lactic acidosis, and most (13/14) had hypoglycemia. Four *FBP1* variants were identified: c.88G > T (p.Glu30Ter), c.652_661delinsTCACGAGGGCT (p.Arg218SerfsTer9), c.960delinsGG (p.Ser321ValfsTer13), and c.902_904del (Glu301del). The c.960delinsGG variant was detected in nine cases, suggesting a founder effect. The c.652_661delinsTCACGAGGGCT is a novel variant. One case had a coexisting partial biotinidase deficiency. Regarding outcome, two patients died during the neonatal period, while the remainder achieved normal neurodevelopment.

**Conclusion:**

This is the first study of FBP1 deficiency in Egypt, which expands the demographic, clinical, and genetic spectrum of this rare disease.

**Supplementary Information:**

The online version contains supplementary material available at 10.1186/s13052-025-02146-w.

## Introduction

**Fig. 1:**
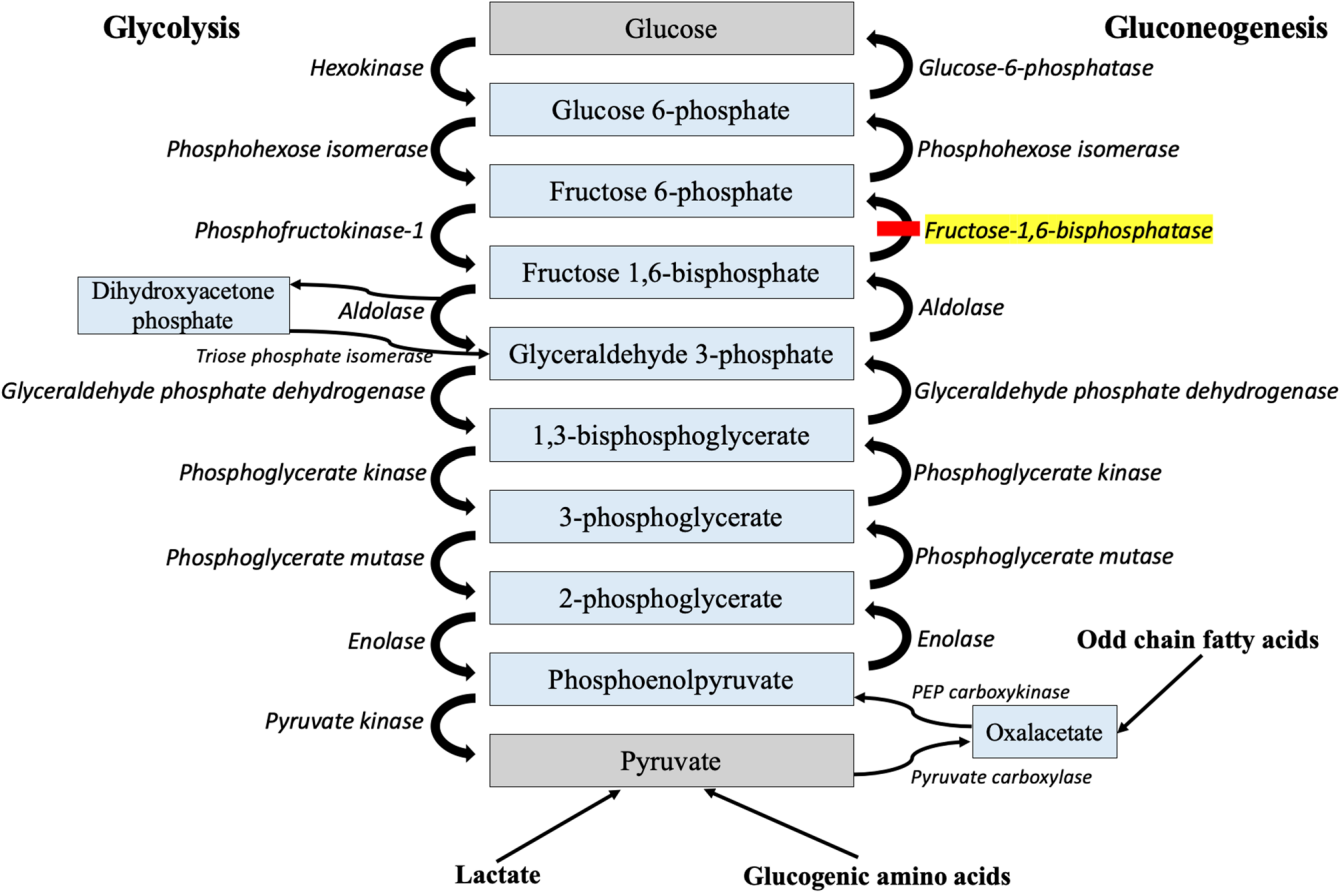
Pathway of gluconeogenesis depicting the defective step in fructose-1,6-bisphosphatase deficiency

Fructose-1,6-bisphosphatase (FBP1; EC 3.1.3.11) deficiency (MIM **#** 229700) is a rare autosomal recessive disorder of gluconeogenesis and fructose metabolism. FBP1 catalyzes the dephosphorylation of fructose-1,6-bisphosphate to fructose-6-phosphate, a key step in hepatic gluconeogenesis (Fig. 1). A deficiency in this enzyme impairs the endogenous formation of glucose from gluconeogenic precursors, such as alanine, lactate/pyruvate, and glycerol [1, 2]. The clinical hallmark of FBP1 deficiency is recurrent episodes of lactic acidosis and ketotic hypoglycemia during early childhood. These are often precipitated by fever, decreased oral intake, or excess fructose consumption. Acute metabolic episodes are commonly associated with encephalopathy, seizures, and elevated liver enzymes [3]. Death may occur secondary to severe, inadequately treated metabolic crisis. In between metabolic crises, patients are typically asymptomatic with normal neurodevelopment, unless prolonged hypoglycemia during acute episodes results in irreversible brain damage [2, 4].

Diagnosis of this disease is confirmed by demonstrating deficient FBP1 enzyme activity or by identifying biallelic pathogenic variants in the *FBP1* gene (MIM * 611570). Human *FBP1* gene (NCBI RefSeq NG_008174.1) is located on chromosome 9q22.2-q22.3, composed of eight exons (NM_000507.4), and encodes a protein of 338 amino acids (NP_000498.2) [3]. Over 100 *FBP1* variants have been reported in association with FBP1 deficiency, some of which show population-specific distributions [4].

To date, more than 150 patients with FBP1 deficiency have been identified worldwide [3]. The estimated prevalence is approximately 1 in 350,000 in the Dutch population and 1 in 900,000 in the French population [5, 6]. The disease may be more prevalent in populations with a high frequency of consanguineous marriage, such as those in Arab countries and Egypt [7–9]. However, no Egyptian cases have been reported thus far.

In this study, we describe—for the first time—a cohort of 14 Egyptian children with FBP1 deficiency. The findings expand the demographic, clinical, and molecular spectrum of FBP1 and potentially enhance early diagnosis and improve clinical outcomes of patients with this rare disorder.

## Patients, materials, and methods

### Study design, setting, and population

This observational study included Egyptian children diagnosed with FBP1 deficiency between 2022 and 2024 at the Pediatric Genetic and Metabolic Unit, Sohag University Hospital (southern Egypt). Diagnosis of FBP1 deficiency was based on typical episodes of hypoglycemia, ketosis, and lactic acidosis, along with biallelic pathogenic or likely pathogenic variants in the *FBP1* gene [3]. The study was approved by the Research Ethics Committee of Sohag Faculty of Medicine (Approval no. Soh-Med-24-05−08PD). Informed consent was obtained from the parents of all participating children. All study procedures were performed in accordance with the ethical principles contained in the 1964 Declaration of Helsinki, and as revised in 2013.

### Clinical assessment

Enrolled children were subjected to comprehensive history taking, physical examination, and developmental evaluation. Collected data included parental consanguinity, family history, sex, current age, age at first presentation, details during acute episodes (e.g., acidotic breathing, altered conscious level, seizures), and outcome. Neurodevelopment was assessed using the Denver II Developmental Screening Test (http://denverii.com). The outcome was categorized as favorable, neurodevelopmental impairment, or death. Details of both emergency and long-term management were also recorded.

### Laboratory evaluation

All participants underwent thorough laboratory investigations upon acute presentations, including blood glucose, lactate, ammonia, arterial blood gas, and urinary ketones. Blood glucose was determined using Cobas C311 Chemistry analyzer system (Roche Diagnostics, Indianapolis, IN, USA), and hypoglycemia was defined at levels less than 45 mg/dl in neonates and less than 50 mg/dl in infants and older children [10, 11]. We measured arterial blood gas and serum lactate using ABL800 FLEX blood gas analyzer (Radiometer Medical ApS, Bronshoj, Denmark), according to manufacturer’s directions. Urinary ketones were evaluated using Combur-Test strips (Roche Diagnostics).

For metabolic screening, dried blood spot samples were collected on Guthrie cards (Whatman 903 filter paper; GE Healthcare Ltd, NJ, USA) and analyzed for acylcarnitine profiles using ACQUITY TQD Tandem Quadrupole UPLC/MS/MS (Waters^®^ Corporation, Milford, MA, USA), following the manufacturer’s instructions. Urinary organic acids were analyzed by gas chromatography-mass spectrometry (GC/MS) using Agilent 7890 gas chromatograph and 5975 mass spectrometer systems (Agilent Technologies Inc., Santa Clara, CA, USA), and results were calculated in µmol per mmol creatinine based on a calibration curve generated from the corresponding organic acid, which was processed under identical conditions.

### Genetic analysis

Genomic DNA was extracted and purified from patients’ peripheral blood samples using the QIAamp DNA Blood Mini Kit (QIAGEN, Germantown, MD, USA). Exome capture was conducted using Nextera Rapid Capture Exome Kit (Illumina Inc., San Diego, CA, USA), covering 214,405 exons (98.3% of exomes in RefSeq database). Sequencing was performed through NovaSeq 6000 systems (Illumina), according to manufacturer’s protocols. FASTQ raw sequencing data were aligned to human genome (reference GRCh37/Hg19) using BWA-MEM algorithm, and variants were called through GATK v.3.5.1 software [12, 13]. Variants were annotated by Ensemble Variant Effect Predictor, followed by filtering and classification using EVIDENCE platform according to the American College of Medical Genetics and Genomics (ACMG) guideline [14, 15]. Only variants relevant to the patient’s clinical phenotype were reported after expert geneticists’ review.

### Data analysis

Data were analyzed using IBM SPSS Statistics for Windows, version 22 (IBM Corp., Armonk, NY, USA). Data were expressed as frequency and percentage or median and interquartile range as appropriate. Due to the small sample size, only descriptive statistics were applied, and no formal hypothesis testing was conducted.

## Results

A total of 14 Egyptian children (10 males, 4 females) from 11 families were diagnosed with FBP1 deficiency at our institution during the period from 2022 to 2024. The patients’ clinical, biochemical, and molecular features are provided in Table 1, and family pedigrees are provided in Additional file 1. Most patients (13/14) had consanguineous parents, and 9/14 had a positive family history, including four with prior sibling deaths. The median age at disease onset was 13 months; most cases (11/14) developed first symptoms between 3 and 24 months of age, while two presented in the first week of life, and one case had a later onset at 36 months of age. Identified triggering factors included fever (6/14) and vomiting/fasting (6/14). All patients exhibited acute metabolic acidosis (pH range 6.9–7.3) along with hyperlactatemia (27–120 mg/dl). Hypoglycemia and ketonuria were detected in 13 and 12 cases, respectively. All experienced altered mental status of variable severity, and almost half (6/14) developed seizures.

**Table 1:** Clinical, biochemical, and molecular features of Egyptian children with fructose-1,6-bisphosphatase deficiency

				First episode												Prognosis		FBP1 variants^#^	
ID^*^	Cons	FH	Sex	Age at onset	Trigg. illness	pH	HCO_3_	BE	Glucose	NH_3_	lactate	Urinary ketones	Altered mental status	Seizure	Enlarged liver	Present age	Outcome		
1	+	+	F	8 m	Fever	6.9	5	−24	30	61	70	4+	+	–	–	5.5 y	Improved	c.960delinsGG (p.Ser321ValfsTer13)	c.960delinsGG (p.Ser321ValfsTer13)
2	+	+	M	2 d	–	6.9	6	−23	36	58	78	4+	+	–	–	Died at 4 d		c.960delinsGG (p.Ser321ValfsTer13)	c.960delinsGG (p.Ser321ValfsTer13)
3	+	+	M	3 m	Vomiting	7.1	8	−17	64	66	120	4+	+	+	–	4.3 y	Improved	c.960delinsGG (p.Ser321ValfsTer13)	c.960delinsGG (p.Ser321ValfsTer13)
4	+	+	F	26 m	Vomiting	7.3	9.8	−16	20	76	69	3+	+	–	+	2.3 y	Improved	c.960delinsGG (p.Ser321ValfsTer13)	c.960delinsGG (p.Ser321ValfsTer13)
5	+	+	F	26 m	Fever	7.1	7.2	−21.1	34	54	27	4+	+	+	–	3.2 y	Improved	c.960delinsGG (p.Ser321ValfsTer13)	c.960delinsGG (p.Ser321ValfsTer13)
6	–	–	M	16 m	Fever	7.0	6.4	−21	44	69	105	4+	+	+	–	2.6 y	Improved	c.960delinsGG (p.Ser321ValfsTer13)	c.88G > T (p.Glu30Ter)
7	+	–	M	14 m	Vomiting, fasting	7.3	10.2	−14	47	72	48	4+	+	+	–	1.6 y	Improved	c.902_904del (p.Glu301del)	c.902_904del (p.Glu301del)
8	+	–	M	9 m	Vomiting	7.1	4.5	−23.5	34	64	63	3+	+	–	+	2.4 y	Improved	c.960delinsGG (p.Ser321ValfsTer13)	c.960delinsGG (p.Ser321ValfsTer13)
9	+	–	M	18 m	Fever	7.2	7	−21	48	75	76	4+	+	+	–	2.5 y	Improved	c.652_661delinsTCACGAGGGCT (p.Arg218SerfsTer9)	c.652_661delinsTCACGAGGGCT (p.Arg218SerfsTer9)
10	+	+	F	24 m	Fasting	7.0	5.7	−21	45	53	90	–	+	–	+	7.6 y	Improved	c.88G > T (p.Glu30Ter)	c.88G > T (p.Glu30Ter)
11	+	+	M	36 m	Fever	7.1	6.8	−24	50	65	140	–	+	–	+	16 y	Improved	c.88G > T (p.Glu30Ter)	c.88G > T (p.Glu30Ter)
12	+	+	M	7 m	Fever	7.0	6	−23	35	54	55	4+	+	–	–	3 y	Improved	c.960delinsGG (p.Ser321ValfsTer13)	c.960delinsGG (p.Ser321ValfsTer13)
13	+	+	M	3 d	–	7.0	7	−23	36	63	110	4+	+	–	–	Died at 6 d		c.960delinsGG (p.Ser321ValfsTer13)	c.960delinsGG (p.Ser321ValfsTer13)
14	+	–	M	12 m	Vomiting, fasting	7.2	7.2	−22	47	76	87	3+	+	+	–	1.6 y	Improved	c.902_904del (p.Glu301del)	c.902_904del (p.Glu301del)

Values for pH, HCO_3_, and BE are shown in mmol/L, glucose and lactate in mg/dL, and NH_3_ is in µg/dL

Abbreviations: *BE* base excess, *Cons* consanguinity, *d* day, *F* female, *FH* family history, *M* male, *m* month, *y* year

* Cases 3 and 4 as well as 10 and 11 are sib pairs, while cases 1 and 2 are cousins

^#^ Described following the Human Genome Variation Society Nomenclature v.21.1.1 (https://hgvs-nomenclature.org) using NCBI Reference Sequence NM_000507.4 (NP_000498.2)

Acylcarnitine profiles were generally unremarkable, except for decreased C2 and C18:2 in Case 5. Abnormal urinary organic acids were observed in three cases: Cases 8 and 9 showed elevated levels of lactic, 3-hydroxybutyric, and 2-hydroxybutyric acids, whereas Case 9 exhibited increased lactic, 3-hydroxyisobutyric, and 2-hydroxyisovaleric acids levels.

Table 2 shows the four *FBP1* variants identified in our study. The most common variant was c.960delinsGG (p.Ser321ValfsTer13), detected in 9 cases (8 homozygous). A novel homozygous variant—c.652_661delinsTCACGAGGGCT—was found in one case. This variant is predicted to be deleterious by Mutation Taster2021, is expected to result in a frameshift at position 218 with premature termination (p.Arg218SerfsTer9), has not been reported in the Genome Aggregation Database (gnomAD) v2.1.1 (https://gnomad.broadinstitute.org/), and is classified as Likely pathogenic per ACMG guidelines [14]. The remaining variants were c.88G >T (p.Glu30Ter) and c.902_904del (Glu301del), identified in three and two cases, respectively.

**Table 2:** *FBP1* variants identified in 14 children with fructose-1,6-bisphosphatase deficiency

Exon	Nucleotide change	Predicted effect	Alleles no.	Mutation Taster2021	gnomAD	dbSNP	ClinVar ID	ACMG class	Reference
Exon 1	c.88G >T	p.Glu30Ter	5	Deleterious	–	rs121918190	870	Pathogenic	[24]
Exon 5	c.652_661delinsTCACGAGGGCT	p.Arg218SerfsTer9	2	Deleterious	–	–	–	Likely pathogenic	This study
Exon 7	c.902_904del	p.Glu301del	4	Deleterious	–	–	–	Likely pathogenic	[1]
Exon 7	c.960delinsGG	p.Ser321ValfsTer13	17	Deleterious	0.0001026	rs1057517733	372,364	Pathogenic	[24]

*FBP1* variants were described following the Human Genome Variation Society Nomenclature v.21.1.1 (https://hgvs-nomenclature.org) using NCBI Reference Sequence NM_000507.4 (NP_000498.2)

A summary of patients’ features by genotype is provided in Additional file 2. Overall, patients with the homozygous c.960delinsGG variant tended to exhibit a more severe phenotype, characterized by earlier age at onset (median 7.5 months), lower blood glucose levels (median 34.5 mg/dL), and higher mortality (2/8). In contrast, the two cases with homozygous c.88G > T variant showed a milder phenotype, with a later age at onset (median 30 months), lower blood lactate levels (median 56 mg/dL), absence of urinary ketones, and no seizures or deaths.

Besides *FBP1* variants, whole-exome sequencing (WES) in Case 7 revealed compound heterozygous variants in the *BTD* gene: NM_001281723.2:c.1336G > C (p.Asp446His; ClinVar ID 1900) and c.517G > A (p.Ala173Thr; ClinVar ID 3898). Quantitative biotinidase enzyme assay showed activity of 14.9 umol/dL/18 h, indicating partial deficiency (Normal reference range > 26; partial deficiency 7–26; and profound deficiency < 7). No remarkable skin or hair abnormalities were observed.

During acute episodes, hypoglycemia was treated with intravenous 10% dextrose, initially administered as 2 mL/kg bolus over 2–5 min, followed by continuous infusion to maintain blood glucose level at the upper normal range, with early transition to enteral feeds as tolerated. Bicarbonate (half calculated dose over 30 min) was administered if pH remained < 7.1, with close monitoring of electrolytes and blood gas to avoid iatrogenic hypernatremia or metabolic alkalosis. Long-term management included prevention of hypoglycemia through avoiding fasting and frequent feeding, proper treatment of acute illnesses that may precipitate metabolic crisis, and restriction of foods and medications that contain fructose, sucrose, glycerol, or sorbitol. Additionally, Case 7 received oral biotin (10 mg/day) for the coexisting biotinidase deficiency.

Time from disease onset to diagnosis varied among cases, ranging from 1 month to 9 years. Case 7 showed a high liability to go through metabolic crisis with any minor illness. Older patients (Cases 10 and 11) demonstrated better metabolic control, tolerating longer fasting without metabolic crises. Regarding outcome, two cases succumbed to death during the neonatal period due to severe metabolic decompensation. The remaining 12 cases showed favorable outcomes with no remarkable neurodevelopmental abnormalities.

## Discussion

This is the first report of FBP1 deficiency in the Egyptian population, presenting a cohort of 14 affected children. Patients generally presented episodes of metabolic acidosis, hypoglycemia, and hyperlactatemia between 3 and 24 months of age, commonly triggered by fever and vomiting/fasting. The most frequently detected *FBP1* variant was c.960delinsGG (p.Ser321ValfsTer13), identified in nine cases. One patient had a coexisting partial biotinidase deficiency. Two patients died during the neonatal period, while the remainder achieved normal neurodevelopment. These findings broaden the demographic, clinical, and genetic spectrum of FBP1 deficiency.

In the current cohort, patients with FBP1 deficiency presented with acute metabolic episodes at a median age of 13 months, which is generally consistent with prior studies [3, 7]. However, only two cases presented during the neonatal period. This contrasts with reports describing neonatal onset, often within the first four days after birth, in about half of affected infants [2, 3, 7, 16, 17]. For instance, Li et al. [17] described that two out of four Chinese patients manifested within the first 72 h of life. Similarly, Emecen Sanli et al. [16] reported neonatal presentation in three out of six patients from Turkey. Moreover, Salih et al. [7] described metabolic episodes within the first day after birth in three out of seven Saudi patients. The small number of cases with FBP1 deficiency identified during the neonatal period in our cohort likely indicates that this genetic disorder has a phenotypic spectrum broader than originally thought, with most subjects not experiencing any metabolic crises before their presenting episode. Future studies measuring additional catabolic markers, such as non-esterified fatty acids and uric acid, may uncover key links between the degree of catabolism and both the age at onset and the severity of clinical presentation.

The majority of patients in the present study exhibited the classic biochemical hallmarks for FBP1 deficiency, including hypoglycemia, hyperlactatemia, metabolic acidosis, and ketonuria. However, two cases showed negative urinary ketone results, highlighting that ketosis, while typical, is not universally present in FBP1 deficiency [2, 3]. Although FBP1 deficiency is usually included in the differential diagnosis of ketotic hypoglycemia with other disorders (e.g., beta-ketothiolase deficiency, pyruvate carboxylase deficiency, respiratory chain defects, and hereditary fructose intolerance), the lack of ketosis may lead to an initial misdiagnosis with non-ketotic hypoglycemic conditions, such as fatty acid oxidation defects and glycogen storage disease type 1 [3, 18]. Regarding blood acylcarnitine profiles, the majority of patients demonstrated no remarkable abnormalities, which is typical for FBP1 deficiency [2, 3]. However, a low C18:2 level was observed in one patient (Case 5). This incidental finding may reflect reduced intake of essential fatty acids, technical or pre-analytical artifacts, or a coexisting inherited metabolic disorder (e.g., carnitine palmitoyltransferase I deficiency) arising from genetic alterations not detectable by standard WES, such as structural rearrangements, deep intronic changes, or regulatory variants.

Urinary organic acid abnormalities were detected in three cases in our cohort, including elevated levels of lactic, 3-hydroxybutyric, 3-hydroxyisobutyric, 2-hydroxybutyric, and 2-hydroxyisovaleric acids. These nonspecific metabolites reflect the build-up of gluconeogenic precursors (e.g., alanine, lactate) and ketone bodies in patients with FBP1 deficiency, particularly during fasting and catabolic states [1, 2]. However, glycerol-3-phosphate, the most characteristic urinary marker of FBP1 deficiency, was not detected in our cohort. Likewise, Salih et al. [7] and Chandrasekhar et al. [19] described cases with FBP1 deficiency who exhibited similar non-specific urinary organic acid abnormalities without detectable glycerol-3-phosphate, while others identified glycerol-3-phosphate in only 50% of patients [20, 21]. This failure of detection may be attributed to the use of conventional GC/MS methods involving solvent extraction, which may miss glycerol-3-phosphate in the presence of high lactate levels [22]. Under such conditions, more sensitive detection of glycerol-3-phosphate is possible using urease pretreatment non-extraction methods [22]. Another possible explanation is the delayed collection of urine samples, as increased urinary glycerol-3-phosphate levels are detectable only during the metabolic crisis and rapidly return to normal once the patient’s condition stabilizes [3, 21]. Given the non-specific clinical features, failure to detect glycerol-3-phosphate can prolong the diagnostic odyssey, which is often ultimately resolved only by next-generation sequencing. To avoid missing this key biochemical marker for FBP1 deficiency, urine samples should be collected as early as possible during acute episodes and analyzed using urease pretreatment non-extraction GC/MS methods [3, 21, 22].

The most frequent *FBP1* variant in our cohort was c.960delinsGG (17 out of 28 alleles; 60%), suggesting a founder effect. It leads to a frameshift and premature stop codon (p.Ser321ValfsTer13) and is classified as pathogenic according to ACMG guidelines. This variant, sometimes denoted as c.960_961insG and c.959dupG, has been described as the most common *FBP1* variant among Japanese populations [23, 24] and has also been reported among patients from France [5], Italy [25], and China [17]. Other *FBP1* variants seem to be more prevalent in certain populations, such as c.841G >A and c.472 C >T among Indians and Pakistanis [20, 26], c.958G >A and c.986T >C in South Brazilians [27], c.114_119dup among Saudis [7], and exon 2 deletion in Turkish [28]. On the other side, our study identified a novel homozygous variant–c.652_661delinsTCACGAGGGCT (p.Arg218SerfsTer9), classified as likely pathogenic [14]. The other two variants (c.88G >T and c.902_904del) detected in our cohort have been reported in patients from Japan and France, respectively [1, 24]. Notably, almost all (13/14) of our patients carried homozygous *FBP1* variants, consistent with the high frequency of consanguineous marriage in the Egyptian population [8].

The dietary management plan for our patients focused on preventing hypoglycemia and acute catabolism through frequent feeding, adequate caloric supplementation during acute illness, and restriction of both fructose and sucrose, particularly in young children. Although there is universal agreement on emergency management, the practice of fructose- and sucrose-restricted regimens varies widely among international centers [29]. In contrast to infants and young children, such dietary restrictions may not be necessary in older patients, as hepatic glycogen reserves increase with age, thereby reducing reliance on gluconeogenesis for maintaining blood glucose [29, 30].

**Fig. 2:**
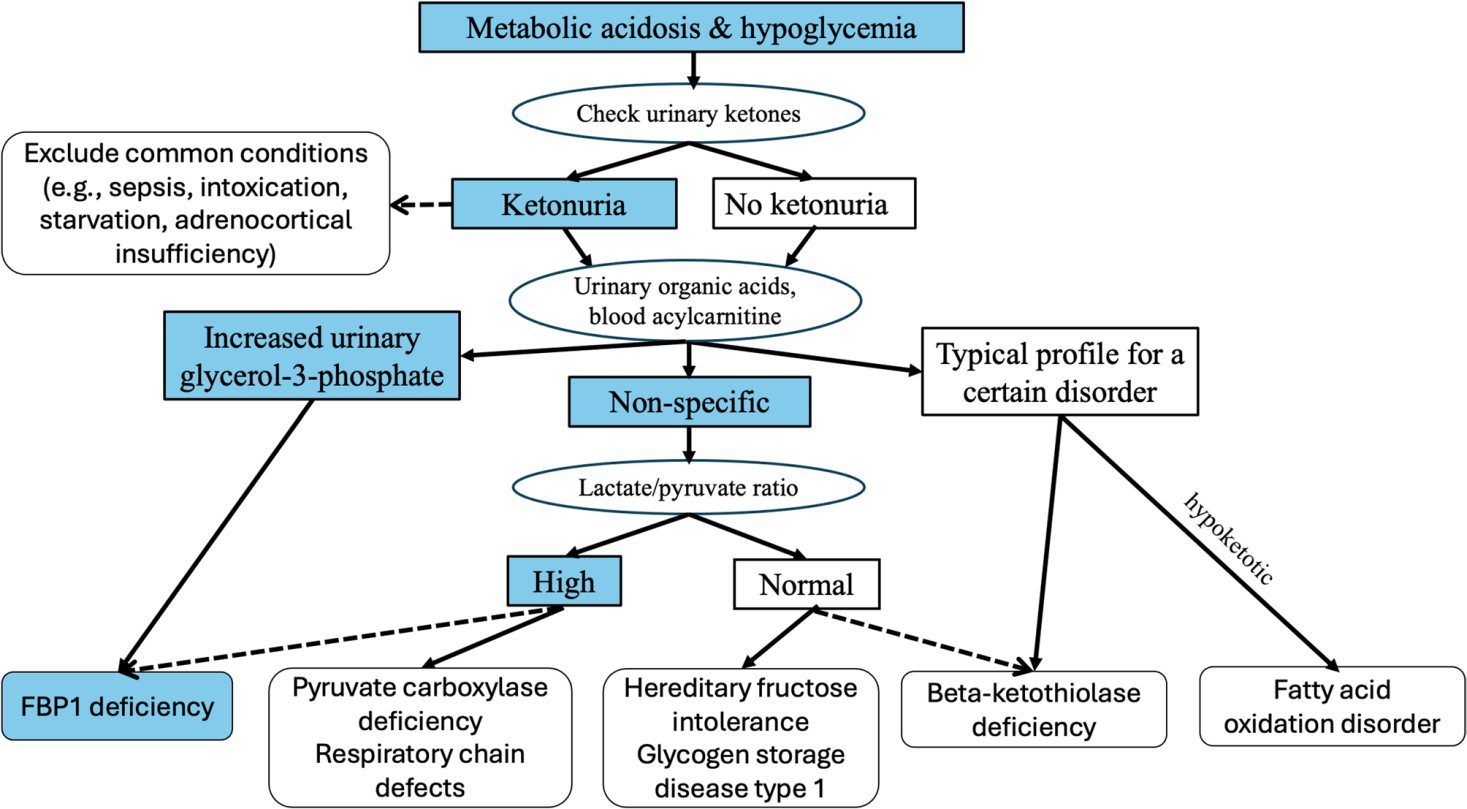
Diagnostic flowchart for fructose-1,6-bisphosphatase (FBP1) deficiency in resource-limited settings

The present cohort revealed a long interval from disease onset to definitive diagnosis, ranging from months to several years, which has also been described in prior reports [7, 21]. Multiple challenges contribute to the delayed or missed diagnosis of FBP1 deficiency, including its nonspecific clinical presentation, lack of physicians’ awareness, limited access to diagnostic facilities in developing countries, and failure to detect urinary glycerol-3-phosphate [3, 21, 22]. A simplified diagnostic flowchart for FBP1 deficiency in resource-limited settings is provided in Fig. 2, but definitive confirmation still requires enzyme assay and/or genetic testing. Description of an increasing number of patients with FBP1 deficiency from different populations helps improve the physicians’ awareness and facilitates timely diagnosis and management of this orphan disorder, which is crucial for better clinical outcomes [20, 21].

The current study represents the first report of FBP1 deficiency (a rare disease) from the Egyptian population, with description of both phenotypic and genetic features. However, we acknowledge some limitations. First, the relatively small sample size restricted the study’s power to conduct formal statistical analyses of the phenotype-genotype associations, calling for larger future studies. Another limitation is the lack of some clinical and laboratory data (e.g., serum pyruvate, non-esterified fatty acids, and uric acid) for metabolic episodes due to incomplete medical records, restricted access to certain diagnostic facilities, and the absence of a national registry for inherited metabolic diseases in Egypt. Finally, the single-center design may limit the generalizability of findings to other geographic regions.

## Conclusion

This study is the first to report FBP1 deficiency in the Egyptian population, describing a cohort of 14 affected children. We identified a likely founder variant (c.960delinsGG), detected in nine cases, and a novel variant (c.652_661delinsTCACGAGGGCT), classified as likely pathogenic. These findings expand the demographic, clinical, and genetic spectrum of FBP1 deficiency and underscore the importance of early diagnosis and treatment.

## Supplementary Information

Additional file 1. Family pedigree charts of study participants

Additional file 2. Genotype-phenotype association in 14 Egyptian children with fructose-1,6-bisphosphatase deficiency

## Data Availability

The data that support the study findings are available from the corresponding author on reasonable request.

## Abbreviations

ACMG: American College of Medical Genetics
GC/MS: Gas chromatography-mass spectrometry
gnomAD: Genome Aggregation Database
FBP1: Fructose-1,6-bisphosphatase
WES: Whole-exome sequencing
