# Supplementary material for: Clinical and molecular characterization of 14 Egyptian children with fructose-1,6-bisphosphatase deficiency
Source: Ital J Pediatr. 2025 Dec 1;51:314. doi: 10.1186/s13052-025-02146-w (PMC12670856; doi:10.1186/s13052-025-02146-w)
Supplement: Supplementary file 1 — Additional file 1. Family pedigree charts of study participants [file 13052_2025_2146_MOESM1_ESM.docx]

**Supplementary material 1**

Family pedigree charts

N

3 yrs

**Family (1)**

5 yrs

4 days case 2

Case 1

5.5 yrs case 1

Case 1

110 yr yrsys

1 yrsys

10 mo

10 yrs

6 yrs

4daysys

4days

**Family (2)**

6.5 yrs

2.25 yrs

Case 4

4.25 yrs

Case 3

**Family (3)**

7 yrs

3.2 yrs

Case 5

2 yrs

**Family (4)**

2.6 yrs

Case 6

**Family (5)**

1.6 yrs

Case 7

**Family (6)**

7.5 yrs

2.4 yrs

Case 8

4 yrs

**Family (7)**

7 yrs

12 yrs

9 yrs

2.5 yrs

Case 9

6 yrs

5 yrs

14 yrs

**Family (8)**

16 yrs

Case 11

10 yrs

17 yrs

7.6 yrs

Case 10

19 yrs

**Family (9)**

7 yrs

4.5 yrs

1.5 yrs

3 yrs

Case 12

10 mo

**Family (10)**

7 mo

6 days

Case 13

4 yrs

**Family (11)**

1.6 yrs

Case 14
