# Supplementary material for: Clinical and molecular characterization of 14 Egyptian children with fructose-1,6-bisphosphatase deficiency
Source: Ital J Pediatr. 2025 Dec 1;51:314. doi: 10.1186/s13052-025-02146-w (PMC12670856; doi:10.1186/s13052-025-02146-w)
Supplement: Supplementary file 2 — Additional file 2. Genotype-phenotype association in 14 Egyptian children with fructose-1,6-bisphosphatase deficiency [file 13052_2025_2146_MOESM2_ESM.docx]

**Additional file 2** Genotype-phenotype association in 14 Egyptian children with fructose-1,6-bisphosphatase deficiency

| **Features** | **c.960delinsGG**  (*n*=8) | **c.88G>T**  (*n*=2) | **c.902_904del**  (*n*=2) | **c.652_661delinsTCACGAGGGCT**  (*n*=1) | **c.960delinsGG / c.88G>T**  (*n*=1) |
| --- | --- | --- | --- | --- | --- |
| Male/female (*n*) | 5/3 | 1/1 | 2/0 | 1/0 | 1/0 |
| Age at onset (median) | 7.5 m | 30 m | 13 m | 18 m | 16 m |
| Lactate (median) | 69.5 | 56 | 67.5 | 76 | 105 |
| Glucose (median) | 34.5 | 47.5 | 47 | 48 | 44 |
| Ketonuria (*n*) | 8/8 | 0/2 | 2/2 | 1/1 | 1/1 |
| Altered mental status (*n*) | 8/8 | 2/2 | 2/2 | 1/1 | 1/1 |
| Seizure (*n*) | 2/8 | 0/2 | 2/2 | 1/1 | 1/1 |
| Enlarged liver (*n*) | 2/8 | 2/2 | 0/2 | 0/1 | 0/1 |
| Mortality (*n*) | 2/8 | 0/2 | 0/2 | 0/1 | 0/1 |

*FBP1* variants are described following the Human Genome Variation Society Nomenclature v.21.1.1 (https://hgvs-nomenclature.org) using NCBI Reference Sequence NM_000507.4.

Values for glucose and lactate are shown in mg/dL

Abbreviations: m, month; *n*, number
